# Understanding university student priorities for mental health and well‐being support: A mixed‐methods exploration using the person‐based approach

**DOI:** 10.1002/smi.3133

**Published:** 2022-02-23

**Authors:** Masha Remskar, Melissa J. Atkinson, Elizabeth Marks, Ben Ainsworth

**Affiliations:** ^1^ Department of Psychology Bath Centre for Mindfulness and Compassion, University of Bath Bath UK

**Keywords:** intervention development, mental health, person‐based approach, university students, well‐being

## Abstract

Poor student well‐being at UK universities is overstretching institutional support services, highlighting a need for effective new resources. Despite extensive literature on mental health and well‐being interventions, students' engagement with support remains unexplored. The study aimed to understand students' experience of engagement with well‐being support, identify their well‐being needs and form concrete recommendations for future intervention design and delivery. The Person‐Based Approach to intervention design was followed to centralise users' experience, in turn maximising acceptability and effectiveness of resources. An online survey (*N* = 52) was followed by three focus groups (*N* = 14). Survey data were analysed descriptively, and reflexive thematic analysis was performed on qualitative data. Mixed‐methods data integration produced four key student priorities for well‐being resources – *ease of access*, *inclusive and preventative approach*, *sense of community and a safe space*, and *applying skills to real‐life contexts*. Five actionable guiding principles for intervention design were produced through consultation with expert stakeholders. This work helps understand why and how students engage with support at university. The resulting recommendations can inform future intervention development, leading to more acceptable, engaging and effective student well‐being resources.

## 1 INTRODUCTION

One in five university students experience mental health concerns, a five‐fold increase over the past decade (Thorley, 2017). Students also report poorer well‐being (i.e., subjective feelings of life satisfaction and fulfilment – a term broader than and distinct from mental health; Barkham et al., 2019; Hughes & Spanner, 2019) than the general population (Insight Network & Dig‐In, 2020). Affected students often underperform or fail academically (Vaez & Laflamme, 2008), or drop out of university altogether (Thorley, 2017), with long‐term implications for achievement, careers and life outcomes (Royal College of Psychiatrists [RCP], 2011). The human cost of student mental health is significant; Student suicide rate of 4.7 per 100,000 equates to 95 deaths every year (Office for National Statistics [ONS], 2018).

Most higher education institutions (HEIs) provide student well‐being support through dedicated welfare teams, and virtually all are reporting an increased demand for well‐being services – six in 10 HEIs reported demand increasing by over 25% within a 5‐year period (Thorley, 2017). With limited funding available (Universities UK [UUK], 2018), universities are struggling to meet these rising demands, leading to routine long waiting times before students can access help (Gallagher, 2014). Failing to address the root causes of poor well‐being and equip students with efficient coping strategies while at university can exacerbate poor mental health (Biasi et al., 2017).

Furthermore, the ongoing COVID‐19 pandemic negatively impacted the university experience, including the provision of teaching and well‐being services (Burns et al., 2020). This coincided with a marked decrease in student well‐being and increased prevalence of clinical‐level depressive symptoms among the group to over 30% (Evans et al., 2021). To break this pattern, policymakers have called for HEIs to make student welfare a strategic priority, emphasising early treatment and prevention of mental illness (House of Commons, 2020; Office for Students [OfS], 2019; UUK, 2015, 2018, 2020). Meta‐analyses suggest that preventative approaches can safeguard against detrimental effects of poor psychological health (Conley et al., 2017; Reavley & Jorm, 2010), however, there is still substantial scope for improved implementation, such as using digital technology to improve access (Conley et al., 2016).

Recent systematic reviews report that a range of psychological interventions can be effective in improving student wellbeing, including cognitive‐behavioural and mindfulness‐based techniques (Breedvelt et al., 2019; Huang et al., 2018; What Works Centre for Well‐being, 2020). However, there is little focus on uptake and effective engagement (i.e., how and why target users engage with the resource; Yardley et al., 2016) – despite insufficient engagement with intervention content reducing the effectiveness of interventions. Student mental health and well‐being interventions often report low engagement across intervention approaches and delivery types, with most distressed individuals even less likely to seek help (the help‐negation effect; Goodwin et al., 2016; Rickwood et al., 2005). Systematic reviews suggest that online resources may improve engagement with student help‐seeking, although note that more high‐quality evidence is needed (Kauer et al., 2014). This highlights the need for better understanding of the student experience and greater attention to the practical side of intervention design, which is crucial for implementation success and effectiveness (Michie et al., 2018). For example, students like online well‐being resources for their accessibility but are sceptical of their potential for communication and human connection (Chan et al., 2016).

In recent decades, the field of behaviour change has recognised the value of target user involvement in developing the most effective and useable interventions (Wicks et al., 2018). The Person‐Based Approach (PBA) to intervention design (PBA; Yardley, Morrison, et al., 2015) is a framework for centralising users' experience throughout planning and development of interventions, via systematic and iterative exploration of target users' perspectives and tailoring of the intervention accordingly. It aims to enhance theory‐ and evidence‐based approaches to intervention development, enabling researchers to understand the users' experience of the intervention in a psychosocial context, anticipate (and pre‐empt) potential barriers to engagement, and improve clarity in the interpretation of outcomes. Resulting alterations make interventions persuasive, feasible and relevant to users (Yardley et al., 2016), translating into greater effectiveness. In practice, adopting the PBA entails a focus on incorporating qualitative or mixed methods research with a representative sample of the target population. In the first instance, an in‐depth understanding of behavioural facilitators and barriers from the users' perspective allows the formation of *guiding principles* – a concise list of key intervention design objectives paired with the design features through which the intervention will meet them (Yardley, Ainsworth, et al., 2015). The guiding principles provide the foundation for further intervention development and evaluation of complex interventions (Medical Research Council [MRC], 2019). The present study exemplifies this initial part of the PBA process.

### 1.1 Research aims

Given the concerns regarding mental health and well‐being at HEIs, there is need for preventative, scalable and student‐accepted approaches to help with student well‐being (UUK, 2018, 2020). In‐depth exploration of how and why students effectively engage with support allows for the design of optimally acceptable and effective future interventions (Yardley, Ainsworth, et al., 2015).

The aim of the present project was to inform the development of interventions improving student well‐being, through understanding students' experience of engagement with well‐being resources, determining students' well‐being needs and establishing guiding principles for the design of student well‐being interventions.

## 2 METHODS

### 2.1 Design

The study utilised a parallel mixed‐methods design. A series of qualitative focus groups was complemented by a predominantly quantitative online survey, then findings from both methodologies combined to produce recommendations for future intervention development. A mixed‐methods approach—previously used in developing well‐being resources tailored to specific populations (e.g., Simpson and Mercer's (2020) mindfulness‐based intervention for people with multiple sclerosis)—provided detailed insight into students' experience through focus groups, paired with the assurance that the identified priorities were echoed by a larger survey sample. Stakeholder consultations were used to ensure that recommendations were appropriate and practical for service developers and providers to implement, in line with best practice for intervention co‐development (Pottie et al., 2021). The research was carried out from a critical realist philosophical position—the researchers presumed that students' priorities for efficient well‐being services exist and can be identified, albeit through the inevitably constructed understanding of social reality (Maxwell, 2012).

### 2.2 Setting

The project was carried out at a research‐intensive HEI based in a mid‐sized city in South‐West England. The institution provides students with varied well‐being support, ranging from group to individual, online and in‐person (pre‐pandemic), grounded in approaches including cognitive behavioural therapy, mindfulness‐based techniques and social activities (e.g., gardening for well‐being; University of Bath, 2020).

### 2.3 Participants

Participants were current students at the University of Bath over 18 years of age, without an existing diagnosis of psychopathology. Advertisement materials inviting students to ‘have their say in shaping well‐being resources’ at their institution were sent through the institution's well‐being services mailing list and the Psychology Department recruitment scheme, totalling 1354 students. The survey recorded 60 responses (4.4% response rate). Initial screening determined that only entries with progress rate over 35% provide usable data, which reduced the sample size to 52 survey participants. Out of this group, 14 participants purposively selected for diversity took part in the three focus groups.

The sample's full demographic and well‐being information is presented in Table 1. All contributors to this research were in full‐time attendance and none had caring responsibilities. The well‐being of survey respondents was moderately good at the time of participation (Tennant et al., 2007). While our sample included a higher proportion of postgraduate students relative to the whole student body (50% vs. 27%, respectively), participants' key demographics (age, ethnicity, gender) reflect the demographic make‐up of the home HEI (University of Bath, 2021) and the region (ONS, 2020), making the sample broadly representative of the wider target population.

**TABLE 1:** Demographics and well‐being assessment of study participants

	Focus group participants (*N* = 14)	Online survey participants (*N* = 52)
Age (years)		
Mean (*SD*)	22.00 (2.45)	21.96 (2.47)
Range	18–28	18–30
Gender *n* (%)		
Female	9 (64.3)	39 (75)
Male	5 (35.7)	12 (23.1)
Other	‐‐	1 (1.9)
Ethnicity *n* (%)		
White	11 (78.6)	36 (69.2)
Asian/Asian British	2 (14.3)	9 (17.3)
Black/Black British	‐‐	1 (1.9)
Hispanic	1 (7.1)	‐‐
Mixed	‐‐	4 (7.7)
Other	‐‐	2 (3.8)
Status *n* (%)		
UG	4 (28.6)	26 (50)
PG	10 (71.4)	26 (50)
Work *n* (%)		
Part‐time	4 (28.6)	21 (40.4)
Not in work	10 (71.4)	31 (59.6)
Past engagement with SS *n* (%)		
Yes	N/A	23 (46.9)^a^
No	N/A	26 (53.1)^a^
Current use of any WB resources *n* (%)		
Yes	6 (42.9)	19 (38.8)^a^
No	8 (57.1)	30 (61.2)^a^
WEMWBS score		
*M (SD)*	N/A	48.67 (8.76)
Range	N/A	25–64
PANAS positive affect score		
*M (SD)*	N/A	31.88 (7.32)
Range	N/A	13–45
PANAS negative affect score		
*M (SD)*	N/A	23.74 (7.74)
Range	N/A	12–49

Abbreviations: PANAS, Positive and Negative Affect Schedule (score range 10–50 for each); PG, postgraduate; SS, University of Bath Student Services; WB, well‐being; WEMWB, Warwick‐Edinburgh Mental Well‐Being Scale (score range 14–70); UG, undergraduate.

^a^ *n* = 49.

### 2.4 Procedure

#### 2.4.1 Survey

Survey data was collected online between 25 June to 5 August 2020, using Qualtrics (2020). The survey consisted of three blocks: demographic information, a well‐being assessment and preferences for new well‐being resources. Questions probing for the impact of COVID‐19 pandemic on well‐being and resource preferences were included to discern general preferences from those arising as a result of this highly unusual context. All responses were anonymous and participants could skip any question they preferred to leave unanswered. On completion, participants were debriefed and signposted to several sources of well‐being support.

#### 2.4.2 Focus groups

Initial plans for three in‐person focus groups were adapted to a virtual format (MS Teams) during the COVID‐19 pandemic. In virtual groups, participants were first reminded of the discussion subject and of their rights, then asked to reiterate informed consent on tape. The discussion was guided by a semi‐structured focus group schedule (see below and supporting Information S1) and moderated by the first author—a female research assistant with methodological experience and interest in the topic. Participants reaffirmed their ongoing consent at the end, as well as having the opportunity to withdraw until the end of the day. Debrief forms with well‐being support contacts were circulated via email following each session. Focus group recordings lasted 34, 58 and 64 min each with three, six and five participants, respectively.

#### 2.4.3 Stakeholder consultations

Data analysis took place after the final focus group (see below). Once the research team were content with preliminary findings (i.e., had a summary of survey data, a probable thematic map and draft guiding principles), the following stakeholders were consulted in individual video call meetings: second and third authors, who are mindfulness intervention experts, the home institution's deputy director of well‐being services and the lead of well‐being support at a neighbouring institute, the University of Bristol. Stakeholders contributed to the iterative development of guiding principles by commenting on the viability of the initial version within the constraints of the well‐being provision system.

### 2.5 Materials and measures

#### 2.5.1 Survey

The Warwick‐Edinburgh Mental Well‐Being Scale (WEMWBS; Tennant et al., 2007) was chosen to gauge participants' current well‐being. WEMWBS is a well‐being questionnaire validated across populations and cultures (Clarke et al., 2011; Taggart et al., 2013) with desirable psychometrics in samples comparable to present one (Cronbach's *α* = 0.89, test‐retest reliability = 0.83 (Stewart‐Brown et al., 2011)). It requires participants to indicate how frequently over the past 2 weeks their well‐being corresponded to each of 14 statements, such as ‘I've been feeling cheerful’, on a scale of 1–5. Scores are summed up in the end, with a higher total implying better mental well‐being (range 14–70).

The Positive and Negative Affect Schedule (PANAS; Watson et al., 1988) assessed tendency to experience negative and positive emotions, as a construct related to well‐being. PANAS is validated in clinical (Ostir et al., 2005) and non‐clinical settings (where Crawford & Henry [2004] report Cronbach's α of 0.89), as well as having good internal and temporal stability (Thompson, 2007). Participants rate, on a scale from 1 to 5, to what extent they felt each of 20 sentiments, such as ‘Enthusiastic’ and ‘Afraid’, over the past 2 weeks. 10 positive and 10 negative items are summed up separately to give scores of positive and negative affect, respectively. Higher scores indicate stronger affect (range 10–50 each).

The survey aimed to assess the key facilitators and barriers relating to both content and delivery of interventions. Students' inclinations towards different types and characteristics of well‐being support offered by the well‐being services were explored using a survey developed for the current study (see supporting Information S1). Participants reported most pressing issues they desired support for, as well as the format of support which would most likely promote their engagement—including group size and composition, delivery format, frequency of sessions and proportion of skill development (vs. didactic) content.

#### 2.5.2 Focus groups

A semi‐structured focus group schedule (see supporting Information S1) was developed to guide the discussions. It consisted of three broad sections: well‐being and its contributing factors, previous engagement with well‐being resources and preferences for future well‐being resources. The questions and prompts were used flexibly, accommodating for the natural progression of conversation and allowing the moderator to follow up points deemed relevant to students' experience and the research objectives.

### 2.6 Data analysis

#### 2.6.1 Survey – Descriptive statistics

Means and standard deviations or frequency counts were described as appropriate for numerical data (i.e., demographics, well‐being scales, resource preferences and topic ratings).

#### 2.6.2 Focus groups – Reflexive thematic analysis

Braun and Clarke's (2006; 2019) reflexive thematic analysis (TA) approach was adopted for analysis of focus group data, maintaining a critical realist stance throughout. Analysis was predominantly data‐driven (i.e., inductive), yet included elements of a deductive approach; Coding itself and construction of themes were inductive, whereas the goals of identifying students' well‐being needs and relating the findings back to efficient well‐being services were set in advance of analysis (i.e., deductively). Combining analytic approaches in this way is recognised and encouraged in reflexive TA (Braun & Clarke, 2020).

Six stages of TA (see Braun & Clarke, 2006, 2014) were followed, even though the process was iterative rather than linear (Braun & Clarke, 2020). This included transcription and initial coding by the first author using NVivo 12 (QSR International, 2020), development of draft themes and subthemes, review and consultation with the final author resulting in theme restructuring, checking new themes against data extracts and refining themes by all authors. Data interpretation was guided by an intervention development framework, the PBA (Yardley, Morrison, et al., 2015), by setting the focus of analysis on practical aspects of students' engagement with well‐being support.

### 2.7 Data integration

Survey and focus group data were first analysed separately, then analytically combined following Guetterman and colleagues' (2015) recommendations for convergent mixed‐methods designs. Qualitative themes identified through reflexive TA served as basis for student priorities because the data set provided sufficient level of detail to establish them. This was then iteratively cross‐referenced with the quantitative data set. Through this process, key student priorities (i.e., those sufficiently present in both data sets) were extracted and interpreted into guiding principles for student well‐being intervention design. Draft guiding principles were reviewed with stakeholders familiar with provision of well‐being support in the HE sector until deemed practically viable (version 3; see also page 7).

Employing multiple data collection methods provided cross‐validation of findings and allowed drawing upon the strengths of each approach; For example, the survey determined the preference that well‐being provision settings limit their group size (i.e., assurance that a finding is echoed by the population). Discussion in the focus group uncovered the reasons behind it and its implications—smaller groups feel less intimidating to students, foster trust and facilitate sharing of personal experience, which is deemed important for efficient participation in well‐being provision (in‐depth understanding of a phenomenon). Finally, consulting stakeholders offered a provider's perspective and ensured that the resulting recommendations were scientifically and practically sound, such as emphasising the balance between available resources and impact of group size on effectiveness (readiness for implementation). A multi‐method approach is essential for nuanced understanding pursued by applied research in complex systems (e.g., Johnson et al., 2017), which includes university‐wide well‐being provision.

## 3 RESULTS

### 3.1 Summary of qualitative findings

**FIGURE 1:**
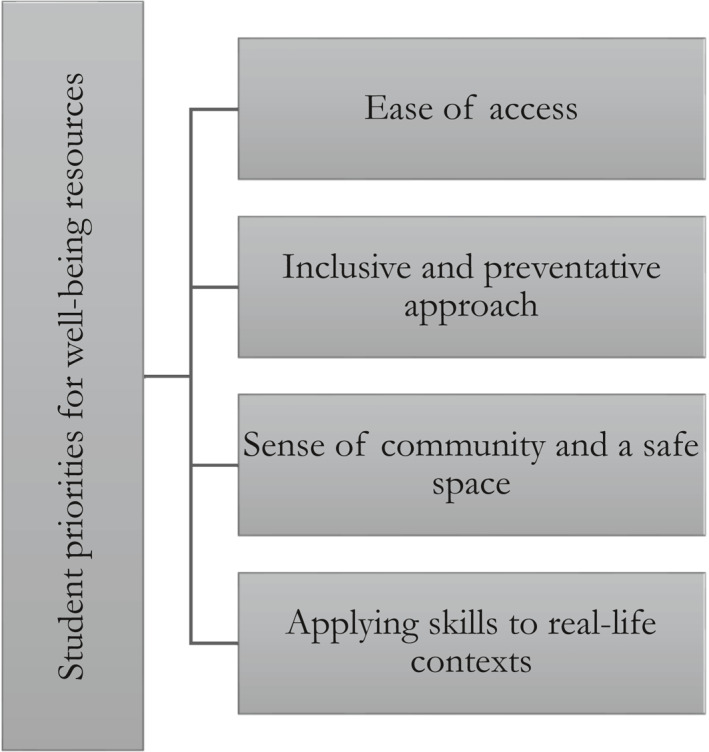
Key student priorities for well‐being resources generated through reflexive thematic analysis (TA) of focus group data

The main four themes generated through reflexive TA of focus group data are presented in Figure 1. Each theme is elaborated on in the following sections.

#### 3.1.1 Theme 1: Ease of access

The first theme captures students' consistent emphasis upon the availability of information regarding well‐being support, as well as support itself. Most participants recognised the need for ease of engagement with resources and described several of the currently experienced barriers to participation.

Some reported being largely unaware of the support available at University, having never engaged with them during their time to date. This suggests that well‐being support presently does not form part of the usual student experience and that the visibility of university services could be improved to raise students' awareness of the available support.

I haven't engaged with the well‐being services, like, at all. I couldn't tell you where they are at uni[versity campus], couldn't tell you what they do, couldn't tell you what they offer. (PC2)

In contrast, others reported previous (or continuing) engagement with well‐being support. When PA3 decided to reach out during lockdown, they found the well‐being advisors to be responsive and helpful. As a result of good accessibility, PA3 has continued engaging with the services.

I liked the‐ ‘cause I contacted them at, like, 2 pm and someone got back to me pretty much straight away, so I felt quite, like, ‘okay, they do, kind of, care. (PA3)

There was a recurrent notion—expressed even by students with positive personal experience—that existing well‐being support for students is scarce and rarely easily accessible. This scarcity creates an atmosphere of competitiveness around accessing mental health help.

They're often, like, under resourced and they're first come, first serve or, like, for those who need it most ‘cause it's not that much, they can't give it to everyone. (PC2)

The idea of inaccessibility was a result of hearsay for some and direct contact with the well‐being team for others. It encompassed practical barriers to participation, such as a prolonged referral process, and inadequate structural support, such as staffing levels. As a result, students were sometimes discouraged from attempting to seek help through the institution.

From what I've heard, um, to get certain support through the university [...] you have to speak to, like, three different people, like, a doctor, then get an interview with someone, then get referred. (PB2)

The approach that we got in our intro[ductory] lecture was kind of like ‘yeah, we're here but we're super understaffed and super overworked so if you're going to try to get a one‐on‐one session you better apply now ‘cause otherwise you're not going to get it ever. (PC5)

Students' feelings of limited support extended beyond initial seeking of help. In practice, support is often restricted to six or eight sessions (University of Bath, 2020) regardless of students' improvement and state of well‐being. This was deemed insufficient by students who had engaged with support, noting that the sudden termination of help left them worried about how they were going to continue looking after their mental well‐being.

Sometimes these 8‐week courses, you know, at the end you just feel like ‘okay, that was really good but now it's gone. (PB3)

In terms of advertising the available support, some felt that other services and aspects of university life were prioritised over their well‐being.

I had no idea that [the Student Services] were in that first introductory lecture that we had – but we had, like, three session on, like, critiquing academic work, so that seems really disproportionate’.ʼ (PC3)

To improve the visibility of well‐being support, students recommended advertising the offer of university services widely and early on in the term. This was expected of the institution as part of looking after their students, as implied by PB4's repeated use of the modal verb should.

Mental health [support] should also be included in the, you know, fresher's week thing. I think straight off the bat students should be offered, um, all the possibilities. (PB4)

Finally, students suggested that minimising practical barriers to participation would stimulate their engagement with well‐being support. Recommended ways of achieving this included a simpler referral process or on‐demand virtual services.

If there was an easy route I would [engage with well‐being resources] but I'm not planning to go out and look for one. (PB2)

Practical aspects of engagement, such as accessibility of the resource, are a common finding in qualitative health intervention literature (O’Connor et al., 2016). Research into barriers to participation for a cancer recovery intervention found that ‘practical barriers’ were the major obstacle participants faced, as described by patients and intervention facilitators (Toivonen et al., 2020). Present findings affirm that availability of the resource is a baseline requirement of successful health interventions, including student well‐being support.

#### 3.1.2 Theme 2: Inclusive and preventative approach

The second theme encompasses the persistent idea that well‐being support caters exclusively to those experiencing severe distress and its consequences for uptake of services. To this end, students desired the reframing of well‐being and its improvement as for everyone, regardless of current mental state. Likewise, participants endorsed expanding the offer of resources focussing solely on well‐being maintenance and advertising them in an inclusive way.

Most participants acknowledged a belief that mental health support at university is limited to those in a particularly bad mental state who need immediate help; that it is *‘for people who need it most’ (PC2)*. This created a notion of a threshold to be reached before one is welcome and able to engage with well‐being support, such as asking oneself *‘Am I really struggling?’ (PB2)* or doubting whether *‘my well‐being is bad enough’ (PC2)*. As a result, students had reservations about seeking help, even if they recognised that they may be able to benefit from it.

I feel there's almost this consensus [...] that only if it's really bad you should go [to the well‐being support team]. (PC4)

The idea of exclusivity of well‐being help was at times amplified by signals that students received from university services themselves.

With the [well‐being] services it always felt—with my panic attacks—that because they weren't influencing my academic work or performance, and because, like, I still was taking care of myself and all that, that they were like ‘okay, so you're fine’ in a sense (PC1)

Certain participants considered well‐being support as *‘a preventative measure’ (PC5)*. They emphasised the idea of building a coping ‘toolkit’ to prepare for inevitable periods of poorer mental well‐being.

When things start getting bad I already, kind of, have a toolkit and a strategy of, like, what am I going to do to get through it instead of, like, hitting rock bottom and then trying to find help. (PC5)

A shift towards a more preventative approach to mental health was welcomed by the majority of participants. An important part of this was the language used in advertising well‐being support to students; participants wished to see resources *‘advertised to everyone’* and coming from a perspective that was *‘not as clinical’ (PC2)*. They theorised that the University adopting this approach would encourage more students to reach out and to do so before their well‐being got severely impaired. For example, one participant felt that the welfare team should *“introduce students to the topic more and to sort of explain that, you know, even if your well‐being is good it's still useful to go.” (PC4)*

Even if it's just a 10 minute chat [...] it's really important that you engage with us even if you feel like you don't necessarily have really bad well‐being […] and ‘we offer these well‐being and mindfulness sessions that [...] can be used as a preventative measure rather than just as a, sort of, treatment measure’ – I think that would be really useful. (PC2)

Nevertheless, participants warned just how deeply embedded the sense of ‘exclusivity’ is, pointing out that some students may still retain the position to only reach out for help when distressed.

For me if it's advertised more it's not going to change anything because I know that I'll only look for one when I know my well‐being is bad. But if it's okay or just slightly bad then I'd rather work on it on my own. (PC4)

Students were unanimous in their belief that presently available support is not sufficiently inclusive, and that this widespread narrative hinders students' help‐seeking. Evidence for a reduction in help‐seeking behaviour as a consequence of socially held beliefs has been demonstrated previously. Evidence suggests that students may be particularly affected by such beliefs, with one systematic review concluding that youth (especially those identifying as male) are disproportionately deterred by stigma from accessing well‐being help (Clement et al., 2015). Indeed, the uncovered normative belief may obstruct students' sense of capability (i.e., being allowed and able to engage with well‐being support), which poses a barrier to successful enactment of this behaviour (Michie et al., 2011). Regardless, the notion is relatively novel, having not been previously identified in relation to inclusivity of services in qualitative literature.

#### 3.1.3 Theme 3: Sense of community and a safe space

The third theme details participants' desire for a supportive environment where people benefit from sharing personal experience. Multiple aspects of the well‐being resource affected this including the setting, process and physical environment where support takes place. As summarised by one of the participants, users *‘want to feel like it's a real safe space’. (PB3)*

Several students stressed that mental health is a very personal subject, so sharing personal experience is challenging. For this reason, students said they only feel comfortable contributing in welcoming environments.

I want to feel like it's a real safe space so, like, everybody going really wants to be there and is, kind of, non‐judgemental. [...] I think that's really important‐ that it’s, yeah, it's clear that this is somewhere where we're all in this together [...] ‘cause, you know, we're all going to bring up sensitive issues, I think, so it needs to be somewhere where people feel very comfortable. (PB3)

Several participants said that a group setting would benefit users over and above particular skill development. A particularly inviting aspect of a group setting was the formation of a community, where members could relate to and learn from others in a similar situation to them.

People are kind of more cautious around attending group things but actually, from experience, people do come along and they get way more out of it just from it being a group experience than they thought that they would. (PA1)

In their approval of group‐based support, students identified key characteristics to improve acceptability. This included having a closed group (committed members who attend regularly) to create a stable environment and building rapport between members.

It's nice to form that kind of community and also feel more comfortable with the people that you're there with. [...] I'd rather have, like, fewer sessions but with the same people rather than it be, like, every week and it be, like, um, bigger or not always the same people. (PB1)

Nearly all participants felt that the sense of community and ‘safe space’ was only possible within small groups. This was underpinned by personal experience of larger groups where the sense of community was lost, and sharing felt less comfortable or even *‘a little scary’ (PB3).*

Students have two distinct, interrelated priorities which should be balanced: a community and a safe space. Relating to others in a similar situation can reduce feelings of isolation with regards to personal struggles (Burlingame et al., 2004). Feeling safe enables the sharing of intimate experiences, which aligns to the capability component of effective behaviour change (Michie et al., 2011). These ideas chime with previous research into therapeutic benefits of groups. Participants of a group therapy for depression reported that a group setting encouraged self‐disclosure and made them feel understood, benefitting well‐being and progress (Schuster et al., 2018).

#### 3.1.4 Theme 4: Applying skills to real‐world contexts

The final theme recounts students' quest for translating the well‐being management skills learned in sessions into real‐life contexts. Specifically, participants wanted more explicit support to bridge this gap, since their needs were not met by current well‐being resources.

A number of students distinguished between in‐session skill practice and translation into daily life. In‐session help was useful but did not guarantee improvements beyond it. PB4 illustrated the mismatch by acknowledging that *‘if you will do this on your own terms is kind of another issue’.* Interventions were more valuable when they explicitly addressed day‐to‐day situations influencing their well‐being.

It's important that it’s about the skills that you pick up – so that I'd know how to, kind of, apply this to the non‐academic work and just anything. (PA3)

Current well‐being resources failed to meet said students' needs when it came to applying skills outside the sessions themselves. Some users felt that several resources (especially mindfulness‐based ones) attempted to do this but lacked clarity on how and why the taught skills could help in daily life.

I had to do a mindfulness thing with a raisin once and it's like ‐ don't tell me to ‘listen to the raisin’, this doesn't really help me with anything. (laughs) You know, if anything it made me more stressed [...] so I'd really appreciate if someone‐ for someone to just really educate me on, you know, what mindfulness actually is and how I can actually use it in, like, a day‐to‐day situation. (PC5)

Practice burden was an issue and participants disclosed that even with resources and instructions for independent practice (e.g., doing mindfulness meditation for 10 min daily), this was not easy to accomplish. Perceived effectiveness of the intervention was diminished as a result. Students wished to see content of the interventions tailored to include help with behaviour change outside session time.

[Changing behaviour] is difficult if you just have a set routine. So it wasn't‐ I don't think [the resource] was very helpful in actually deterring the bad habits that could‐ that actually contribute to our, I guess, bad mood. (PB4)

Participants' ability to transfer the newly acquired well‐being skills to everyday situations is vital for long‐term sustained change in the face of limited availability of support (Gallagher, 2014). Furthermore, quantitative evidence on effective student mental health interventions indicates that skill‐oriented resources were seven times more likely to produce clinically significant improvements in anxiety, depression and emotional well‐being compared to information only (Conley et al., 2013). This provides a moral and economic case for expanding the skill‐oriented content of well‐being resources over and above students' recommendations.

### 3.2 Online survey: Resource preferences

Participants' interest and format preferences are summarised in Table 2. Students predominantly reported being interested in the described resource (see discussion for how this may have been impacted by contextual factors). In terms of specific preferences for the resource in development, survey respondents' views largely aligned with focus group participants. Students preferred a blended approach (i.e., combined in‐person and virtual settings) and online workshops over those taking place in‐person. This was possibly due to current COVID‐related concerns. A majority of participants voted for small or medium group settings, as seen in Theme 3. The sentiment was not as clear – nor as strong – with regards to group composition. Participants wished to receive support relatively frequently, either weekly or fortnightly. Finally, respondents mirrored the desire of focus group participants for predominantly skill‐based resources (Theme 4).

**TABLE 2:** Preferences for a group well‐being resource expressed in the survey

	Participant preferences (*N* = 48)
Interest *n* (%)	
Definitely interested	14 (29.2)
Somewhat interested	30 (62.5)
Not interested	4 (8.3)
Format *n* (%)	
Blended	26 (55.3)^a^
Online	13 (27.7)^a^
In person	8 (17.0)^a^
Group size *n* (%)	
Small (up to 20)	30 (62.5)
Medium (up to 50)	10 (20.8)
Large (up to 100)	1 (2.1)
Do not mind	7 (14.6)
Group composition *n* (%)	
UG/PG only	7 (14.6)
Mixed students	16 (33.3)
Mixed students and staff	9 (18.8)
Do not mind	16 (33.3)
Frequency of sessions *n* (%)	
Weekly	18 (37.5)
Fortnightly	20 (41.7)
Monthly	9 (18.8)
Other	1 (2.1)
Proportion skill‐based	
Mean (SD)	65.74% (15.43%)
Median	70%

Abbreviations: PG, postgraduate; UG, undergraduate.

^a^ *n* = 47.

### 3.3 Guiding principles for student well‐being interventions

The survey and focus groups provided an understanding of students' priorities for engagement with well‐being support at university. From this, the research team followed the PBA to highlight main *behavioural issues* standing in the way of students seeking support (Table 3). These provided a basis for *key design objectives*, which are met with recommendations for *intervention features* (Yardley, Ainsworth, et al., 2015). The translation into guiding principles integrated the present projects' multi‐methods results, existing literature and consultations with stakeholders familiar with well‐being service provision (see also Data integration section).

**TABLE 3:** Behavioural issues students face when engaging with university well‐being support

Key behavioural issues
1. Students find well‐being support at universities hard to access and insufficient for their needs.
2. Students feel that well‐being support available through universities is aimed at (and exclusively available to) those experiencing severe distress.
3. Students do not acknowledge that mental health and well‐being require (or can benefit from) maintenance, instead only seeking support when the situation is serious.
4. Students feel unable to meaningfully engage with well‐being support that feels impersonal and does not offer a supportive (figurative and physical) environment.
5. Students do not engage with well‐being support that cannot be easily applied to real‐life issues.

For instance, the issue of ‘students find[ing] well‐being support at universities hard to access and insufficient for their needs’ (*behavioural issue 1*) has been identified both in our data and in previous work (Burns et al., 2020; Thorley, 2017). It was thus translated into a recommendation that interventions in this context should aim ‘to improve accessibility of WB support for university students’ (*key design objective 1*). We further suggested ways of meeting the objective by ‘provid[ing] content through widely accessible means (e.g., online or “blended” approach)’ or ‘mak[ing] information on WB support more readily available (e.g., include leaflets in fresher's week activities, send out weekly/fortnightly emails to all students)’ (*intervention features 1*). In contrast, the authors were not aware of any previous findings of ‘students feel[ing] that well‐being support available through universities is aimed at (and exclusively available to) those experiencing severe distress’ (*behavioural issue 2*). This was discussed at length by our qualitative sample, as well as welcomed by the expert stakeholder panel, so we deemed it significant enough to translate into corresponding *design objectives* and *intervention features*. This process was repeated for all behavioural issues listed in Table 3. The resulting guiding principles for the development of student well‐being resources are summarised in Table 4.

**TABLE 4:** Guiding principles for the development of student well‐being interventions (version 3)

Key design objectives	Intervention features
To improve accessibility of WB support for university students.	⁃ Provide content through widely accessible means (e.g., online or ‘blended’ approach) ⁃ Make information on WB support more readily available (e.g., include leaflets in fresher's week activities, send out weekly/fortnightly emails to all students)
To challenge the belief that WB support is aimed only at those who struggle.	⁃ Ensure students are aware of preventative WB support (vs. only high intensity/clinical) ⁃ Minimise practical barriers to participation (e.g., make self‐referral sufficient)
To present WB as a state to be maintained rather than ‘fixed’ when poor.	⁃ Advertise the importance of WB maintenance for health and academic outcomes ⁃ Stress the benefits of early and preventative engagement with WB support (i.e., the idea of building a coping ‘toolkit’ in advance)
To provide a supportive environment conducive to sharing personal experience.	⁃ Employ teaching tactics to build community within larger groups (e.g., breakout rooms) ⁃ Ensure the physical environment sessions are held in is comfortable and relaxing (if delivered in person)
To offer WB support acknowledging and directly addressing vday‐to‐day issues.	⁃ Explicitly apply WB skills (e.g., mindfulness, compassion) to real‐life contexts in‐session ⁃ Frame the intervention itself and its content as skill‐based and relevant to common student concerns ⁃ Offer clear guidance and support about integrating skills into daily life during sessions.

Abbreviation: WB, well‐being.

## 4 DISCUSSION

The present study informs the development of student well‐being interventions. A qualitative exploration of students' well‐being needs identified four core priorities for effective resources: *Ease of access, inclusive and preventative approach, community and a safe space,* and *applying skills to real‐life contexts*. Along with findings of a broader quantitative survey and consultations with stakeholders, the findings were distilled into a set of recommendations for intervention development. Such concise and concrete guidelines will guide future intervention design to make student well‐being interventions for acceptable and efficient. The PBA framework ensured that the process of producing the guidelines was systematic and centred around the target group's experience, enhancing its validity and the credibility of outcomes.

The context in which research is carried out inevitably shapes its conclusions (Phillippi & Lauderdale, 2018). Present research was conducted early on during the COVID‐19 pandemic, Most participants indicated that their well‐being was negatively impacted by the pandemic, which is in line with existing research on this (Evans et al., 2021). As a result, opinions on well‐being support were altered – for example, over half of all survey respondents reported increased interest in well‐being support. There was also greater emphasis on physical safety (i.e., minimising the risk of transmission), which was not a concern in previous research. The pandemic may have further influenced the findings in more subtle ways. Lockdown measures may have made practical aspects of engagement, such as *ease of access*, more salient—in addition to general scarcity of well‐being support, there was now an added element of physical inaccessibility for all non‐virtual help. Finally, participants' desire for a *sense of community* could have been magnified during a period of its distinct lack, when many experienced social isolation (Banerjee & Rai, 2020).

However, it is unlikely that conclusions would have been drastically different in the absence of the pandemic, since several ideas resonate with previous literature (including accessibility and community mentioned above; Burlingame et al., 2004; O’Connor et al., 2016). Finally, while preferences for future resources may have been swayed by the present context, past experience that students discussed mostly took place pre‐pandemic. The fact that both narratives told a similar story reassures those seeking to learn from the findings for a different (post‐pandemic) context.

### 4.1 Strengths, limitations and future directions

Present research is methodologically robust – qualitative exploration of students' experience provided an in‐depth insight into engagement with well‐being help at HEIs. Integrating this with a broader survey assessment, despite its relatively small sample size, enabled a more wholesome evaluation of student well‐being needs than a purely qualitative study, since qualitative research does not (intend to) provide generalisability of findings (Silverman, 2013). Thus, the study added to the limited literature—exploring motives behind students' engagement, rather than quantitatively evaluating intervention effects with little consideration of why those effects were (or were not) present.

The adoption of the PBA, a renowed intervention development framework (Yardley, Ainsworth, et al., 2015), enhanced the work's methodological rigour. It provided a blueprint for exploring the target group's well‐being needs, ensuring that the process was systematic and detailed. It also mandated the translation of findings into ready‐to‐use guidelines, increasing the project's impact potential. Nevertheless, the PBA is designed as an iterative process, whereas the present study only consulted the target group once. Future research can build on this foundation by producing a prototype intervention based on the guiding principles and continuing the approach.

In addition, the PBA requires a representative sample of the whole target user population for input, which this study cannot claim to have achieved. Our sample only included students without current psychological diagnoses. This constricts the generalisability of findings (i.e., our guiding principles) to low‐intensity well‐being support but is less applicable to acute psychological services—the need for which unfortunately also increased due to the pandemic (Evans et al., 2021).

Finally, the study consulted students as one homogenic group, which does not reflect the group's diverse reality (Higher Education Statistics Agency [HESA], 2020). It did not recruit a significant number of students who may be structurally predisposed to experiencing poorer well‐being (e.g., Black, Asian, and minority ethnic [BAME] or lesbian, gay, bisexual or transgender [LGBT+] students; OfS, 2019) and whose circumstances differ from those of most students (e.g., part‐time students, those with caring responsibilities). While the findings present a valuable starting point—given the paucity of research on the subject—future studies should aim to distinguish student well‐being needs and priorities for different demographic profiles and circumstances. To achieve this, studies could focus on hard‐to‐reach student groups and tailor their approach accordingly (Ellard‐Gray et al., 2015).

## 5 CONCLUSION

The present mixed‐method investigation determined student priorities for acceptable and efficient well‐being support at university. Several of its conclusions further existing findings, whereas other ideas are novel – most notably, students' assertion of the need for an inclusive and preventative approach to well‐being, which has so far only been expressed by service providers (rather than users). The findings themselves—particularly distilled into guiding principles (see Table 4)—are direct recommendations for more efficient future student well‐being support. This work amplifies the calls for a sector‐wide shift towards a more systemic and preventative management of student well‐being (e.g., Hughes & Spanner, 2019). Formalised findings and the engagement of stakeholders in the process contribute towards a stronger, more legitimate case for this. By adopting the PBA, current findings were elevated above the passive speculation that research outcomes are too often confined to; Instead, they were translated into the ‘language of intervention and implementation’ (Sandelowski & Leeman, 2012, p. 1404), which lends itself to application and promises tangible impact.

## CONFLICT OF INTEREST

Authors declare no conflicts of interest.

## ETHICS STATEMENT

University of Bath's Psychology Research Ethics Committee approved this study (#20–167).

## AUTHOR CONTRIBUTIONS

Masha Remskar, Melissa J. Atkinson, Elizabeth Marks, Ben Ainsworthconceived the project idea. Masha Remskar developed the research aims and materials, gained ethical approval, conducted the focus groups, analysed the data, consulted stakeholders, drafted the guiding principles and this manuscript. Ben Ainsworth provided guidance on design, data collection, data interpretation and the formation of guiding principles. Melissa J. Atkinson and Elizabeth Marks gave feedback on study design, materials and initial results. Stakeholders Melissa J. Atkinson, Elizabeth Marks, Anthony Payne, Andrew Ayers and Nicola Taylor helped the translation of findings into guiding principles. Elizabeth Marks, Melissa J. Atkinson and Ben Ainsworth reviewed this manuscript.

## Supporting information

Supporting Information S1

Supporting Information S2

## Data Availability

Supplementary materials are available through the University of Bath Research Data Archive and accessible at https://doi.org/10.15125/BATH‐00987.
