# Supplementary material for: Understanding university student priorities for mental health and well‐being support: A mixed‐methods exploration using the person‐based approach
Source: Stress Health. 2022 Feb 23;38(4):776–89. doi: 10.1002/smi.3133 (PMC9790713; doi:10.1002/smi.3133)
Supplement: Supplementary file 1 — Supporting Information S1 [file SMI-38-776-s001.docx]

**Online survey content**

*Note: Survey was hosted on platform Qualtrics.*

**Block 1 – Well-being assessment**

**THE WARWICK-WDINBURGH MENTAL WELL-BEING SCALE (WEMWBS)**

Below are some statements about feelings and thoughts. Please tick the box that best described your experience if each over the last 2 weeks.

*Options: 1 – None of the time, 2 – Rarely, 3 – Some of the time, 4 – Often, 5 – All of the time*

1. I’ve been feeling optimistic about the future.
2. I’ve been feeling useful.
3. I’ve been feeling relaxed.
4. I’ve been feeling interested in other people.
5. I’ve had energy to spare.
6. I’ve been dealing with problems well.
7. I’ve been thinking clearly.
8. I’ve been feeling good about myself.
9. I’ve been feeling close to other people.
10. I’ve been feeling confident.
11. I’ve been able to make up my own mind about things.
12. I’ve been feeling loved.
13. I’ve been interested in new things.
14. I’ve been feeling cheerful.

*Citation: Warwick-Edinburgh Mental Well-being Scale (2006). NHS Scotland, University of Warwick and University of Edinburgh.*

What factors were most detrimental to your mood and well-being over the two weeks?

*(text box)*

What factors tend to be detrimental to your mood and well-being generally?

*(text box)*

**POSITIVE AND NEGATIVE AFFECT SCHEDULE (PANAS-SF)**

Indicate the extent you have felt this way over the past week.

*Options: 1 – Very slightly or not at all, 2 – A little, 3 – Moderately, 4 – Quite a bit, 5 – Extremely*

1. Interested
2. Distressed
3. Excited
4. Upset
5. Strong
6. Guilty
7. Scared
8. Hostile
9. Enthusiastic
10. Proud
11. Irritable
12. Alert
13. Ashamed
14. Inspired
15. Nervous
16. Determined
17. Attentive
18. Jittery
19. Active
20. Afraid

*Citation: Watson, D., Clark, L. A., & Tellegen, A. (1988). Development and validation of brief measures of positive and negative affect: the PANAS scales. Journal of Personality and Social psychology, 54(6), 1063.*

In your opinion, has the current context of COVID-19 affected your well-being over the past two weeks? If so, how?

*(text box)*

**Block 2 – Engagement with well-being resources**

Are you currently using any well-being resources with the intention of improving your mood and well-being?

- Yes
- No

If yes, please specify format and name resource.

*(text box)*

The University’s Wellbeing Services are collaborating with the Bath Centre for Mindfulness and Compassion to develop a new mindfulness-based well-being resource for its staff and students. Present plans include a short series of in-person workshops providing information and active skill practice, giving attendees tools to put into practice in their daily life. Workshops will be delivered once monthly to mixed groups of up to 20 students and staff of the University of Bath, each lasting 50 minutes. Based on the above description, how likely would you be interested in engaging with such a resource?

*(1 – Not at all interested, 2 – Somewhat interested, would attempt engagement, 3 – Certainly interested, would engage with the resource)*

In your opinion, is the current context of COVID-19 influencing your interest in the new well-being resource? If so, how?

*(text box)*

**Block 3 – Exploration of delivery preferences**

Which **format of delivery** would make the new well-being resource the best suited to your needs and preferences?

- In-person workshop
- Online workshop
- A combination of both
- Other – please specify

Which **group size** would make the new well-being resource the best suited to your needs and preferences?

- Small (up to 20)
- Medium (up to 50)
- Large (up to 100)
- Very large (more than 100)
- I don’t mind

Which **group composition** would make the new well-being resource the best suited to your needs and preferences?

- Undergraduate or postgraduate students only
- Mixed undergraduate or postgraduate students
- Mixed students and staff
- I don’t mind
- Other – please specify

Which **frequency of sessions** would make the new well-being resource the best suited to your needs and preferences?

- Once weekly
- Once fortnightly
- Once monthly
- Once termly
- Other – please specify

What proportion of the workshops would you like to be **skills-based** versus information and background teaching?
*(Sliding scale 0 – 100)*

Currently there is evidence that a range of mindfulness-based workshops can have a benefit on people’s well-being, and we are considering offering each workshop around particular themes. Please rate on a scale of 0 – 5 how interested you would be in the following themes:

Introduction to mindfulness and present moment awareness

Relating differently to thoughts and feelings

Using mindfulness in movement and daily life

Managing high standards and self-criticism

Kindness and compassion

Gratitude, joy and thanksgiving

Reducing loneliness and building connection

*(Sliding scales 1-5 for each, where 1 – not at all interested, 5 – extremely interested)*

In addition to the above, are there any sessions you would be particularly interested in attending? Please specify the topics of interest below.

*(Text box)*

**Block 4 – Demographics**

Role within the University of Bath:

- Undergraduate student
- Postgraduate student

Mode of attendance:

- Full-time
- Part-time

University course/programme: *(text box)*

Age: *(text box)*

Gender:

- Female
- Male
- Other
- Prefer not to say

Ethnicity:

- Asian / Asian British
- Black / African / Caribbean / Black British
- White / White British
- Mixed / Multiple ethnic groups
- Other ethnic group (please specify)

Background status:

- Home/UK
- EU
- International

Employment status alongside studies:

- Not employed
- Employed part-time
- Employed full-time

Caring responsibilities:

- No caring responsibilities
- Caring responsibilities for a child
- Caring responsibilities for an adult

The University wishes to include people who would potentially use the well-being resource (such as yourself) in its development in order to make it the most user-friendly and effective. As part of this, we will be running an online) focus group in the near future. If you are interested in being contacted about taking part in this focus group, please leave your university email below.

Rest assured that this information will be stored securely and entirely separately from all the answers you have provided in this survey and will be used ONLY to invite you to the online focus group. **Providing your email does not oblige you to participate, it only means that you may be invited to partake.** You can still decline the invitation or withdraw your participation at any time.

*(text box for email)*
