# Supplementary material for: Understanding university student priorities for mental health and well‐being support: A mixed‐methods exploration using the person‐based approach
Source: Stress Health. 2022 Feb 23;38(4):776–89. doi: 10.1002/smi.3133 (PMC9790713; doi:10.1002/smi.3133)
Supplement: Supplementary file 2 — Supporting Information S2 [file SMI-38-776-s002.docx]

**Focus Group Schedule**

**Session outline**

1. Introductions, restating of aim and plan
2. Read through admin:
   1. Voluntary contribution and right to withdraw
   2. Recording
   3. Confidentiality and anonymity
3. Questions before we begin?

----- start recording -----

1. Go round, say name, state consent
2. Discussion
3. Anything to add, comment on, questions?
4. Anyone wish to withdraw contribution?

----- stop recording -----

1. Any questions off record, clarifications regarding research
2. Thanks, free to contact research team, goodbye

**Focus group schedule**

1. First of all, how is everyone doing? We are living in a strange time and all of our lives have changed drastically in a very short amount of time. Even before this, going to university comes with many perks but also many challenges, which continuously affect out well-being. How is your well-being currently?
2. What factors do you currently see as being supportive to your well-being and why?
3. What factors do you currently see as being detrimental to your well-being and why?
4. Who has previously or is currently engaging with any well-being resources, provided by the University or otherwise?
5. What did you like about the resources you engaged/are engaging with and why?
6. What did you dislike about the resources you engaged/are engaging with and why?

“The University’s Wellbeing Services are collaborating with the Bath Centre for Mindfulness and Compassion to develop a new mindfulness-based well-being resource for its students. Present plans include a short series of in-person workshops providing information and active skill practice, giving attendees tools to put into practice in their daily life. Workshops are intended to be delivered once fortnightly, in medium to large groups, each lasting 50 minutes.”

1. Would you be interested in engaging with this kind of resource?
2. What format and content would your ideal well-being resource include?

Delivery format

Group size

Number and duration of sessions

1. What topics would you be interested in learning about in relation to mindfulness and why?

Introduction to mindfulness and present moment awareness

Relating differently to thoughts and feelings

Using mindfulness in movement and daily life

Managing high standards and self-criticism

Kindness and compassion

Gratitude, joy and thanksgiving

Reducing loneliness and building connection

1. It is good practice in the health sector to evaluate the efficiency of resources and interventions. If the organisers of the workshops included short well-being questionnaires at the first and last well-being session (much like the ones you filled out in Part 1 of this study), would you be willing to complete them as part of engagement with the well-being resource? This is providing that your data would be handled in compliance to very strict Data Management policies set by the University.
2. Is there anything else anyone wishes to add, anything the University should be aware of in relation to its well-being services? Anything particular to the new resource we’re developing? Anything at all?
